# Transcatheter Aortic Valve Replacement for Bicuspid vs. Tricuspid Aortic Stenosis among Patients at Low Surgical Risk in China: From the Multicenter National NTCVR Database

**DOI:** 10.3390/jcm12010387

**Published:** 2023-01-03

**Authors:** Nanchao Hong, Wenzhi Pan, Xianbao Liu, Daxin Zhou, Jianan Wang, Junbo Ge

**Affiliations:** 1 Department of Cardiology, Zhongshan Hospital, Shanghai Institute of Cardiovascular Diseases, Fudan University, 180 Fenglin Road, Shanghai 200032, China; 2 National Clinical Research Center for Interventional Medicine, Shanghai 200032, China; 3 Department of Cardiology, Second Affiliated Hospital, Zhejiang University School of Medicine, Hangzhou 310009, China

**Keywords:** transcatheter aortic valve replacement, bicuspid aortic stenosis, low surgical risk, trans-carotid access

## Abstract

Background: This study aims to compare the outcomes of transcatheter aortic valve replacement (TAVR) with self-expandable valves for bicuspid aortic valve (BAV) vs. tricuspid aortic valve (TAV) stenosis patients who are at low surgical risk. Methods: Participants were enrolled from 36 centers in China between January 2017 and December 2021. The primary endpoint event was all-cause mortality and all stroke at 30 days. Results: Among 389 patients at low surgical risk that underwent TAVR, 229 patients were BAV stenosis (mean age, 72.9 years; 65.1% men). There was no significant difference in the rate of all-cause death between two populations at 30 days. However, the rate of all stroke was significantly higher in the BAV group at 30 days (3.3% vs. 0%; odds ratio (OR), 0.97 (95% confidence interval (CI), 0.94 to 0.99); *p* = 0.044). By multivariate logistic regression analysis, trans-carotid access was associated with a higher all stroke rate at 30 days (OR, 29.20 (95% CI, 3.97 to 215.1); *p* = 0.001). Conclusions: In this national registry-based study, patients treated for BAV vs. TAV stenosis had no significant difference in all-cause mortality at 30 days, but trans-carotid access was associated with a higher all stroke rate after TAVR at 30 days.

## 1. Introduction

Since Cribier and colleagues reported the first case of transcatheter aortic valve replacement (TAVR) in 2002 [1], TAVR has undergone an accelerated period of development, changing the treatment of patients with aortic valve stenosis (AS). Randomized controlled trials have demonstrated the safety, efficacy and feasibility of TAVR, first in inoperable, and then in high surgical risk, intermediate, and recently low surgical risk patients [2,3,4,5,6]. However, these trials excluded bicuspid anatomy. The increasing experience of the operator and refinements in the technology have led to progressive reductions in the incidence of adverse events associated with TAVR, and there are increasingly bicuspid aortic valve (BAV) patients undergoing TAVR treatment.

Compared with Western countries, Chinese AS patients have the characteristics of a high proportion of BAV and high calcium burden [7,8]. Of patients undergoing surgery for aortic stenosis, 15% to 29% have been reported to have BAV [9,10]. An analysis from the western registry showed that about 3.2% of all TAVR procedures in the United States were performed for BAV [11]. However, BAV accounted for nearly half of all TAVR patients in China [12,13], which was associated with early degeneration leading to AS or aortic regurgitation [14]. Previously published studies on the outcomes of TAVR in BAV were limited to a small sample size [15], to older patients who were at higher surgical risk [11], or to those with a balloon-expandable valve [16]. Given that BAV was frequently present in younger and low surgical risk AS patients, prognostic data from TAVR treatment of BAV stenosis was critical to direct the treatment of these patients who are at low surgical risk. 

The National Transcatheter Valve Therapeutics Registry (NTCVR) database was co-initiated by National Clinical Research Center for Interventional Medicine and Chinese Cardiovascular Association, which is the largest database that includes TAVR procedures performed in China [17]. In this national registry-based study, we aimed to compare the outcomes of TAVR with self-expandable transcatheter heart valves (THVs) for BAV vs. tricuspid aortic valve (TAV) stenosis patients who are at low surgical risk. 

## 2. Materials and Methods

### 2.1. Study Population

**Figure 1:**
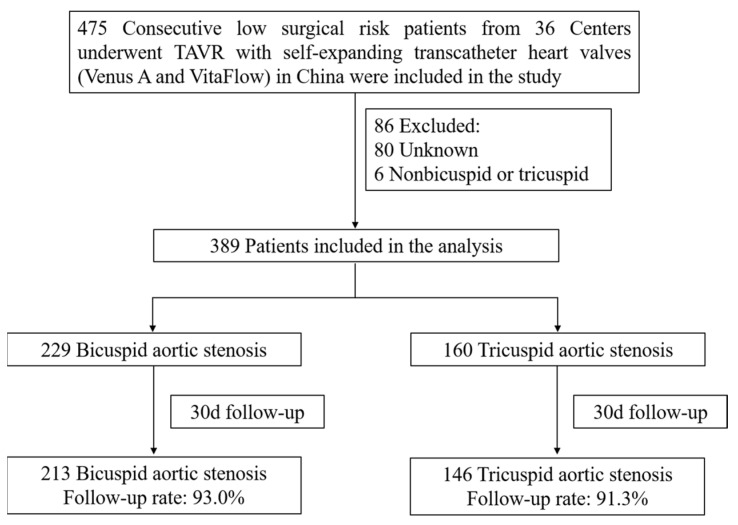
The design of the study.

All consecutive patients undergoing TAVR with self-expandable THVs (Venus A [Venus MedTech, Hangzhou, China] and VitaFlow [MicroPort, Shanghai, China]) from 36 centers in China between January 2017 and December 2021 were evaluated in this study (Figure 1). Baseline characteristics, procedural details, and clinical outcomes were prospectively collected, while the analysis was performed retrospectively. The study included all severe symptomatic AS patients at low surgical risk undergoing TAVR. Low surgical risk was defined as having an STS predicted risk of mortality (STS-PROM) score of less than 4%. Severe AS was defined by echocardiographic criteria including a mean gradient > 40 mmHg (1 mmHg = 0.133 kPa) or peak jet velocity > 4.0 m/s and aortic valve area < 1.0 cm^2^. 

### 2.2. Procedures and Outcomes

Self-expandable THVs were used in all severe AS patients, including two domestic self-expanding THVs: Venus A (Venus Medtech Inc., Hangzhou, China) and the VitaFlow valve (Shanghai MicroPort CardioFlow Medtech Co., Ltd., Shanghai, China). Venus A and Vitaflow were the first-generation self-expanding THVs in China [18]. We performed a post-balloon dilation if a moderate to severe aortic valve regurgitation or high transvalvular gradient existed. The primary endpoint event was all-cause mortality and all stroke at 30 days. The study was conducted in accordance with the Declaration of Helsinki and approved by the Ethics Committee of the Zhongshan Hospital, Fudan University, China. 

### 2.3. Statistical Analysis

All statistical analyses were performed using SPSS version 25 (SPSS, Inc., an IBM Company, Chicago, IL, USA). Categorical data are presented as the number of events and frequencies, and continuous data are presented as mean ± SD. Comparisons between the two groups were performed using the Chi-square test or Fisher’s exact test for categorical variables, and independent *t*-tests for continuous covariates. To identify independent predictors of all stroke after TAVR, candidate variables for the multivariable logistic regression model were required to have clinical relevance and a *p*-value < 0.05 in the univariate analysis, which included all available baseline clinical, echocardiographic, and procedural data. All *p* values were two-sided, and *p* < 0.05 was considered significant for all tests.

## 3. Results

### 3.1. Patient Population

Between January 2017 and December 2021, a total of 475 patients at low surgical risk who underwent TAVR for aortic stenosis with self-expandable THVs at 36 institutions in China were included in the primary analysis. Some 86 patients were excluded because of unknown anatomy or other unknown reasons. A total of 389 patients at low surgical risk (229 with BAV and 160 with TAV) were included in the final analysis (Figure 1). 

Baseline characteristics of the BAV group and TAV group are shown in Table 1. Compared with the TAV group, the rates of NYHA functional class III or IV, incidences of diabetes mellitus, prior myocardial infarction, prior percutaneous coronary intervention (PCI), and pre-existing permanent pacemakers (PPM) in BAV patients were significantly lower, and BAV patients were also younger than TAV patients. As for pre-procedural imaging analysis (echocardiographic and coronary CT), the aortic valve area of BAV patients was significantly smaller than TAV patients, and the percentages of leaflet calcification and aortic insufficiency were higher in the BAV group (Table 2).

### 3.2. Procedural and Clinical Outcomes

Procedural details are presented in Supplementary Table S1. There were no significant differences in procedure status, procedure access, balloon pre-dilation rate, type of anesthesia and the method of vascular closure between the two groups (all *p* > 0.05). 

Clinical outcomes are shown in Table 3. There was no significant difference in the rates of all-cause death between the BAV and TAV group at discharge (0% vs. 0%) or 30 days (0.9% vs. 0.7%; odds ratio (OR), 1.37 (95% confidence interval (CI), 0.12 to 15.3); *p* = 1.00). There was no significant difference in the rates of all-cause death or stroke between the BAV and TAV group at 30 days (4.2% vs. 0.7%; OR, 6.40 (95% CI, 0.80 to 51.05); *p* = 0.053). However, the rate of all stroke was significantly higher in the BAV group both at discharge (2.6% vs. 0%; OR, 0.97 (95% CI, 0.95 to 0.99); *p* = 0.045) and at 30 days (3.3% vs. 0%; OR, 0.97 (95% CI, 0.94 to 0.99); *p* = 0.044). In the secondary clinical outcomes, there were no significant differences in the rates of major vascular complications, major bleeding, myocardial infarction, new-onset atrial fibrillation, new PPM implantation, or moderate/severe paravalvular leak (PVL) between the two groups, both at discharge and at 30 days (all *p* > 0.05).

### 3.3. Multivariable Analysis

A total of 359 patients (213 with BAV and 146 with TAV) completed a 30-day follow-up. The rate of all stroke in the entire population was 1.9%. The candidate variables for the univariate logistic regression analysis for predictors of all stroke are shown in Table 4. Candidate variables for the multivariable model were required to have clinical relevance and a *p*-value < 0.05 in the univariate analysis. Interestingly, trans-carotid access and BAV were predictors of all stroke on univariate analysis. However, on multivariate logistic regression analysis, the BAV was not significantly different (Table 4). By multivariate analysis, trans-carotid access was the only independent predictor of all stroke at 30 days (OR, 29.20 [95% CI, 3.97 to 215.1]; *p* = 0.001).

## 4. Discussion

In this national registry-based study of AS patients at low surgical risk who had undergone TAVR with self-expandable THVs in China, the primary results were as follows: (1) BAV patients had a low 30-day all-cause mortality (0.9%); (2) compared with TAV patients, BAV patients had higher rates of in-hospital and 30-day all stroke. However, there were no significant differences in all-cause mortality, the rates of major vascular complications, major bleeding, myocardial infarction, new-onset atrial fibrillation, new PPM implantation, or moderate/severe PVL between BAV and TAV patients both at discharge and at 30 days; (3) by multivariate logistic regression analysis, trans-carotid access was associated with a higher all stroke at 30 days after TAVR.

This is the first report from the NTCVR database on outcomes of TAVR in patients at low surgical risk in China. There are limited randomized clinical trials of TAVR vs. surgical aortic valve replacement (SAVR) for the treatment of BAV stenosis. Low surgical risk patients with BAV stenosis are often considered more suitable for SAVR. In the present study, BAV patients account for 58.9% of all low surgical risk TAVR patients, which was significantly higher than a recent study based on the western registry [16]. The present study included 389 patients at low surgical risk who underwent TAVR; in 229 BAV stenosis patients (mean age, 72.9 years; 65.1% men; mean STS 2.6%), the rates of NYHA functional class III or IV, incidences of diabetes mellitus, prior myocardial infarction, prior PCI and pre-existing PPM of BAV patients were significantly lower than TAV patients, which may be explained by the younger age of BAV patients. BAV patients demonstrated a 30-day all-cause mortality of 0.9%. Other secondary clinical outcomes were low and comparable to the TAV group. In addition, these outcomes were comparable with published surgical clinical trials on BAV stenosis and reported 30-day mortality ranging from 0.4% to 2.5% [19,20,21,22,23]. However, the 30-day all stroke rates of BAV patients were significantly higher than TAV patients in the present study, and were also higher than the outcomes of SAVR and TAVR with a balloon-expandable valve for TAV stenosis [6], but were comparable to the results of a recent systematic review which analyzes the clinical outcomes of TAVR in BAV patients [24]. 

Due to the superficial and less calcified carotid arteries, trans-carotid TAVR is an easier technique compared with trans-femoral TAVR. It has also been shown that trans-carotid TAVR has similar clinical outcomes compared with the trans-femoral TAVR regarding mortality and morbidity [25,26]. However, the preoperative evaluation of the cerebrovascular anatomy is crucial for trans-carotid TAVR, and previous studies showed that patients with ≥50% carotid stenosis or presence of a plaque are at a high risk for embolization [27,28]. The insertion of large sheaths and the increase in the time of hypoperfusion through the circle of Willis could potentially increase the risk of stroke in trans-carotid TAVR [29]. In this study, we found that trans-carotid access and BAV were predictors of all stroke on univariate analysis, but trans-carotid access was the only independent predictor of all stroke at 30 days after TAVR by multivariate logistic regression analysis (OR, 29.20 [95% CI, 3.97 to 215.1]; *p* = 0.001). The lack of cerebral protection during TAVR may contribute to the increased risk of all stroke in patients with trans-carotid TAVR in our study. 

For BAV stenosis patients with low surgical risk, the selection of TAVR or SAVR should be carefully considered combined with the anatomical suitability, coronary artery access for future interventions, feasibility of repeated TAVR or SAVR, pacemaker incidence, and valvular thrombosis to optimize the lifetime management of treatment. Although the rate of 30-day all stroke was significantly higher in the BAV group, this difference disappeared after multivariate logistic regression analysis in the present study. In the future, prospective randomized clinical studies comparing TAVR vs. SAVR with long-term follow-up data are needed to guide the optimal management of BAV stenosis in young low surgical risk patients.

## 5. Conclusions

In this national registry-based study, patients receiving TAVR with self-expandable valves treated for BAV vs. TAV stenosis had no significant difference in all-cause mortality at 30 days. However, the rate of all stroke was significantly higher in the BAV group at 30 days. By multivariate logistic regression analysis, trans-carotid access was associated with a higher all stroke rate after TAVR at 30 days. 

## Figures and Tables

**Table 1:** Baseline Characteristics of Low-Risk Patients.

	BAV(*n* = 229)	TAV(*n* = 160)	*p* Value
Age, yrs	72.9 ± 6.9	75.3 ± 6.7	0.0007
Male	65.1% (149/229)	66.9% (107/160)	0.711
NYHA functional class III or IV	57.2% (131/229)	68.8% (110/160)	0.021
STS-PROM score *, %	2.6 ± 0.9	2.5 ± 1.0	0.652
Smoking	21.0% (48/229)	26.9% (43/160)	0.175
Diabetes mellitus	13.5% (31/229)	23.8% (38/160)	0.009
Hypertension	44.1% (101/229)	52.5% (84/160)	0.220
Hyperlipidemia	15.3% (35/229)	13.1% (21/160)	0.551
Peripheral vascular disease	3.5% (8/229)	5.0% (8/160)	0.462
Prior CVA/TIA	6.1% (14/229)	8.1% (13/160)	0.442
Chronic lung disease	8.7% (20/229)	10.6% (17/160)	0.532
Prior myocardial infarction	0.4% (1/229)	4.4% (7/160)	0.020
Prior PCI	4.4% (10/229)	9.4% (15/160)	0.047
Pre-existing PPM	0% (0/229)	2.5% (4/160)	0.028
Atrial fibrillation/flutter	6.6% (15/229)	10.0% (16/160)	0.216
Carcer	3.9% (9/229)	2.5% (4/160)	0.627

Values are mean ± SD or n/N (%). * The STS-PROM score estimates the rate of death at 30 days among patients undergoing SAVR on the basis of a pre-defined number of baseline demographic and clinical characteristics and procedural variables. NYHA = New York Heart Association; STS-PROM = Society of Thoracic Surgeons Predicted Risk of Mortality; TIA = Transient ischemic attack; CVA = Cerebrovascular accident; PCI = Percutaneous coronary intervention;; PPM = Permanent pacemaker.

**Table 2:** Pre-Procedural Imaging Analysis.

	BAV(*n* = 229)	TAV(*n* = 160)	*p* Value
Echocardiographic			
LVDD (mm)	47.8 ± 11.0	50.5 ± 9.7	0.016
Mean gradient (mmHg)	67.1 ± 21.6	64.2 ± 22.0	0.210
Aortic valve area (cm^2^)	0.5 ± 0.3	0.6 ± 0.2	0.010
LVEF, %	56.9 ± 11.9	58.4 ± 9.9	0.219
Aortic insufficiency	79.9% (183/229)	90.6% (145/160)	0.004
Mitral insufficiency	81.7% (187/229)	84.4% (135/160)	0.485
Tricuspid insufficiency	74.2% (170/229)	72.5% (116/160)	0.703
Coronary CT			
Leaflet calcification	97.8% (224/229)	92.5% (148/160)	0.023
Annulus calcification	57.6% (132/229)	48.1% (77/160)	0.064
Annulus area (mm^2^)	456.5 ± 117.4	458.4 ± 101.5	0.870
Annulus circumference (mm)	79.5 ± 32.6	79.1 ± 36.7	0.919

Values are mean ± SD or n/N (%). LVDD = Left ventricular end diastolic diameter; LVEF = Left ventricular ejection fraction.

**Table 3:** Clinical Outcomes in the Low-Risk Group.

	BAV(*n* = 229)	TAV(*n* = 160)	*p* Value
In-hospital outcomes			
All-cause death	0% (0/229)	0% (0/160)	-
All stroke	2.6% (6/229)	0% (0/160)	0.045
All-cause death or stroke	2.6% (6/229)	0% (0/160)	0.045
Major vascular complications	3.1% (7/229)	3.1% (5/160)	1.000
Major bleeding	2.6% (6/229)	1.3% (2/160)	0.566
MI	0.9% (2/229)	0.6% (1/160)	1.000
New-onset atrial fibrillation	2.2% (5/229)	1.9% (3/160)	1.000
New-onset LBBB	10.0% (23/229)	11.3% (18/160)	0.703
New-onset AVB	7.4% (17/229)	8.1% (13/160)	0.799
New PPM implantation	6.6% (15/229)	8.8% (14/160)	0.416
Moderate/severe PVL	2.6% (6/229)	2.5% (4/160)	1.000
30-day outcomes			
Follow-up rate	93.0% (213/229)	91.3% (146/160)	0.521
All-cause death	0.9% (2/213)	0.7% (1/146)	1.000
All stroke	3.3% (7/213)	0% (0/146)	0.044
All-cause death or stroke	4.2% (9/213)	0.7% (1/146)	0.053
Major vascular complications	3.3% (7/213)	3.4% (5/146)	1.000
Major bleeding	4.7% (10/213)	2.1% (3/146)	0.304
MI	0.9% (2/213)	0.7% (1/146)	1.000
New-onset atrial fibrillation	2.8% (6/213)	2.7% (4/146)	1.000
New-onset LBBB	12.7% (27/213)	12.3% (18/146)	0.922
New-onset AVB	8.5% (18/213)	8.9% (13/146)	0.881
New PPM implantation	10.3% (22/213)	12.3% (18/146)	0.554
Moderate/severe PVL	3.8% (8/213)	2.7% (4/146)	0.820

Values are n/N (%). MI = Myocardial infarction; LBBB = Left bundle branch block; AVB = Atrioventricular block; PPM = Permanent pacemaker; PVL = paravalvular leak.

**Table 4:** Univariate and Multivariable Predictors of All Stroke After TAVR.

Variables	Univariate Analysis				Multivariate Analysis			
	OR	Lower 95% CI	Upper 95% CI	*p*-Value	OR	Lower 95% CI	Upper 95% CI	*p*-Value
Age	1.033	0.926	1.153	0.556	-	-	-	-
Female	0.766	0.147	4.004	1.000	-	-	-	-
NYHA III or IV	0.454	0.100	2.057	0.434	-	-	-	-
STS score	1.131	0.501	2.551	0.767	-	-	-	-
Smoking	0.557	0.066	4.687	1.000	-	-	-	-
Diabetes mellitus	1.881	0.357	9.899	0.361	-	-	-	-
Hypertension	2.806	0.538	14.639	0.265	-	-	-	-
Hyperlipidemia	0.991	0.117	8.390	1.000	-	-	-	-
Peripheral vascular disease	0.981	0.968	0.995	1.000	-	-	-	-
Prior CVA/TIA	2.282	0.265	19.672	0.398	-	-	-	-
Chronic lung disease	1.652	0.193	14.120	0.496	-	-	-	-
Prior myocardial infarction	0.982	0.968	0.995	1.000	-	-	-	-
Prior PCI	2.486	0.288	21.492	0.374	-	-	-	-
Pre-existing PPM	0.982	0.969	0.995	1.000	-	-	-	-
Atrial fibrillation/flutter	0.980	0.966	0.995	1.000	-	-	-	-
Carcer	0.981	0.968	0.995	1.000	-	-	-	-
LVDD	0.960	0.877	1.050	0.368	-	-	-	-
Mean gradient at basement	1.004	0.973	1.037	0.784	-	-	-	-
AVA by echo base	7.268	0.174	303.859	0.298	-	-	-	-
LVEF	1.019	0.946	1.098	0.624	-	-	-	-
Aortic insufficiency	0.457	0.087	2.409	0.302	-	-	-	-
Leaflet calcification	0.279	0.032	2.453	0.284	-	-	-	-
Transcarotid access	30.160	4.685	194.15	0.006	29.200	3.965	215.06	0.001
Balloon pre-dilatation	1.020	1.005	1.035	1.000	-	-	-	-
BAV	1.032	1.008	1.056	0.045	-	-	-	0.995

NYHA = New York Heart Association; TIA = Transient ischemic attack; CVA = Cerebrovascular accident; PCI = Percutaneous coronary intervention; PPM = Permanent pacemaker; LVDD = Left ventricular end diastolic diameter; AVA = Aortic valve area; LVEF = Left ventricular ejection fraction; BAV = Bicuspid aortic valves.

## Data Availability

The data presented in this study are available on request from the corresponding author.

## Supplementary Materials

The following supporting information can be downloaded at: https://www.mdpi.com/article/10.3390/jcm12010387/s1, Table S1: Procedural Details.

## References

[1] Cribier A., Eltchaninoff H., Bash A., Borenstein N., Tron C., Bauer F., Derumeaux G., Anselme F., Laborde F., Leon M.B. (2002). Percutaneous Transcatheter Implantation of an Aortic Valve Prosthesis for Calcific Aortic Stenosis. Circulation.

[2] Leon M.B., Smith C.R., Mack M.J., Miller D.C., Moses J.W., Svensson L.G., Tuzcu E.M., Webb J.G., Fontana G.P., Makkar R.R. (2010). Transcatheter aortic-valve implantation for aortic stenosis in patients who cannot undergo surgery. N. Engl. J. Med..

[3] Smith C.R., Leon M.B., Mack M.J., Miller D.C., Moses J.W., Svensson L.G., Tuzcu E.M., Webb J.G., Fontana G.P., Makkar R.R. (2011). Transcatheter versus surgical aortic-valve replacement in high-risk patients. N. Engl. J. Med..

[4] Leon M.B., Smith C.R., Mack M.J., Makkar R.R., Svensson L.G., Kodali S.K., Thourani V.H., Tuzcu E.M., Miller D.C., Herrmann H.C. (2016). Transcatheter or surgical aortic-valve replacement in intermediate-risk patients. N. Engl. J. Med..

[5] Thourani V.H., Kodali S., Makkar R.R., Herrmann H.C., Williams M., Babaliaros V., Smalling R., Lim S., Malaisrie S.C., Kapadia S. (2016). Transcatheter aortic valve replacement versus surgical valve replacement in intermediate-risk patients: A propensity score analysis. Lancet.

[6] Mack M.J., Leon M.B., Thourani V.H., Makkar R., Kodali S.K., Russo M., Kapadia S.R., Malaisrie S.C., Cohen D.J., Pibarot P. (2019). Transcatheter aortic-valve replacement with a balloon-expandable valve in low-risk patients. N. Engl. J. Med..

[7] Pan W.Z., Li M.F., Zhou D.X., Guan L.H., Cheng L.L., Ge J.B. (2015). Prevalence and echocardiographic feature of bicuspid aortic valve in patients with severe aortic stenosis: An echocardiography database analysis. Zhonghua Xin Xue Guan Bing Za Zhi.

[8] Jilaihawi H., Wu Y., Yang Y., Xu L., Chen M., Wang J., Kong X., Zhang R., Wang M., Lv B. (2015). Morphological characteristics of severe aortic stenosis in China: Imaging corelab observations from the first Chinese transcatheter aortic valve trial. Catheter. Cardiovasc. Interv..

[9] Husso A., Airaksinen J., Juvonen T., Laine M., Dahlbacka S., Virtanen M., Niemelä M., Mäkikallio T., Savontaus M., Eskola M. (2021). Transcatheter and surgical aortic valve replacement in patients with bicuspid aortic valve. Clin. Res. Cardiol..

[10] Elbadawi A., Saad M., Elgendy I.Y., Barssoum K., Omer M.A., Soliman A., Almahmoud M.F., Ogunbayo G.O., Mentias A., Gilani S. (2019). Temporal trends and outcomes of transcatheter versus surgical aortic valve replacement for bicuspid aortic valve stenosis. JACC Cardiovasc. Interv..

[11] Halim S.A., Edwards F.H., Dai D., Li Z., Mack M.J., Holmes D.R., Tuzcu E.M., Thourani V.H., Harrison J.K., Brennan J.M. (2020). Outcomes of transcatheter aortic valve replacement in patients with bicuspid aortic valve disease: A report from the Society of Thoracic Surgeons/American College of Cardiology Transcatheter Valve Therapy Registry. Circulation.

[12] Liu X.B., Jiang J.B., Zhou Q.J., Pu Z.X., He W., Dong A.Q., Feng Y., Jiang J., Sun Y., Xiang M.X. (2015). Evaluation of the safety and efficacy of transcatheter aortic valve implantation in patients with a severe stenotic bicuspid aortic valve in a Chinese population. J. Zhejiang Univ. Sci. B.

[13] Liao Y.B., Li Y.J., Xiong T.Y., Ou Y.W., Lv W.Y., He J.L., Li Y.M., Zhao Z.G., Wei X., Xu Y.N. (2018). Comparison of procedural, clinical and valve performance results of transcatheter aortic valve replacement in patients with bicuspid versus tricuspid aortic stenosis. Int. J. Cardiol..

[14] Masri A., Svensson L.G., Griffin B.P., Desai M.Y. (2017). Contemporary natural history of bicuspid aortic valve disease: A systematic review. Heart.

[15] Forrest J.K., Ramlawi B., Deeb G.M., Zahr F., Song H.K., Kleiman N.S., Chetcuti S.J., Michelena H.I., Mangi A.A., Skiles J.A. (2021). Transcatheter aortic valve replacement in low-risk patients with bicuspid aortic valve stenosis. JAMA Cardiol..

[16] Makkar R.R., Yoon S.H., Leon M.B., Chakravarty T., Rinaldi M., Shah P.B., Skipper E.R., Thourani V.H., Babaliaros V., Cheng W. (2021). Association Between Transcatheter Aortic Valve Replacement for Bicuspid vs Tricuspid Aortic Stenosis and Mortality or Stroke Among Patients at Low Surgical Risk. JAMA.

[17] Hong N., Pan W., Zhou D., Ge J. (2022). The China Heart Valve Center and National Transcatheter Valve Therapeutics Registry database. Cardiol. Plus.

[18] Zhou D., Pan W., Wang J., Wu Y., Chen M., Modine T., Mylotte D., Piazza N., Ge J. (2020). VitaFlow transcatheter valve system in the treatment of severe aortic stenosis: One-year results of a multicenter study. Catheter. Cardiovasc. Interv..

[19] Borger M.A., Preston M., Ivanov J., Fedak P.W., Davierwala P., Armstrong S., David T.E. (2004). Should the ascending aorta be replaced more frequently in patients with bicuspid aortic valve disease?. J. Thorac. Cardiovasc. Surg..

[20] Girdauskas E., Disha K., Borger M.A., Kuntze T. (2014). Long-term prognosis of ascending aortic aneurysm after aortic valve replacement for bicuspid versus tricuspid aortic valve stenosis. J. Thorac. Cardiovasc. Surg..

[21] Itagaki S., Chikwe J.P., Chiang Y.P., Egorova N.N., Adams D.H. (2015). Long-term risk for aortic complications after aortic valve replacement in patients with bicuspid aortic valve versus marfan syndrome. J. Am. Coll. Cardiol..

[22] Andrei A.C., Yadlapati A., Malaisrie S.C., Puthumana J.J., Li Z., Rigolin V.H., Mendelson M., Clennon C., Kruse J., Fedak P.W. (2015). Comparison of outcomes and presentation in men-versus-women with bicuspid aortic valves undergoing aortic valve replacement. Am. J. Cardiol..

[23] Holmgren A., Enger T.B., Näslund U., Videm V., Valle S., Evjemo K.J., Friberg Ö., Wahba A. (2021). Long-term results after aortic valve replacement for bicuspid or tricuspid valve morphology in a Swedish population. Eur. J. Cardiothorac. Surg..

[24] Chen C.H., Jiang H., Martin O., Wilson-Smith A.R. (2022). Procedural and clinical outcomes of transcatheter aortic valve replacement in bicuspid aortic valve patients: A systematic review and meta-analysis. Ann. Cardiothorac. Surg..

[25] Overtchouk P., Modine T. (2018). A comparison of alternative access routes for transcatheter aortic valve implantation. Expert. Rev. Cardiovasc. Ther..

[26] Kirker E.B., Hodson R.W., Spinelli K.J., Korngold E.C. (2017). The carotid artery as a preferred alternative access route for transcatheter aortic valve replacement. Soc. Thorac. Surg..

[27] Overtchouk P., Folliguet T., Pinaud F., Fouquet O., Pernot M., Bonnet G., Hubert M., Lapeze J., Claudel J.P., Ghostine S. (2019). Transcarotid approach for transcatheter aortic valve replacement with the sapien 3 prosthesis: A multicenter French registry. JACC Cardiovasc. Interv..

[28] Praz F., Wenaweser P. (2018). Transcatheter aortic valve replacement via the transcarotid access. Circ. Cardiovasc. Interv..

[29] Mylotte D., Sudre A., Teiger E., Obadia J.F., Lee M., Spence M., Khamis H., Al Nooryani A., Delhaye C., Amr G. (2016). Transcarotid transcatheter aortic valve replacement: Feasibility and safety. JACC Cardiovasc. Interv..

