# Supplementary material for: Transcatheter Aortic Valve Replacement for Bicuspid vs. Tricuspid Aortic Stenosis among Patients at Low Surgical Risk in China: From the Multicenter National NTCVR Database
Source: J Clin Med. 2023 Jan 3;12(1):387. doi: 10.3390/jcm12010387 (PMC9821306; doi:10.3390/jcm12010387)
Supplement: Supplementary file 1 [file jcm-12-00387-s001.zip › jcm-2093862-supplementary.pdf]

Supplementary Table S1. Procedural Details

	BAV(*n*=229)	TAV(*n*=160)	*P* value
Procedure status			
Elective	99.1% (227/229)	100% (160/160)	0.514
Emergent	0.9% (2/229)	0% (0/160)	0.514
Transfemoral access	95.2% (218/229)	98.7% (158/160)	0.103
Transcarotid access	2.6% (6/229)	1.3% (2/160)	0.566
Transapical access	2.2% (5/229)	0% (0/160)	0.081
Balloon pre-dilatation	92.6% (212/229)	92.5% (148/160)	0.977
Type of anesthesia			
General anesthesia	93.4% (214/229)	93.1% (149/160)	0.900
Local anesthesia	6.6% (15/229)	6.9% (11/160)	0.900
Vascular closure way			
Arterial closure device	79.5% (182/229)	74.4% (119/160)	0.237
Surgical sutures	20.5% (47/229)	25.6% (41/160)	0.237

Values are n/N (%).
